# Changes in workplace car parking and commute mode: a natural experimental study

**DOI:** 10.1136/jech-2018-210983

**Published:** 2018-10-03

**Authors:** Craig S Knott, Stephen J Sharp, Oliver T Mytton, David Ogilvie, Jenna Panter

**Affiliations:** MRC Epidemiology Unit, Centre for Diet and Activity Research (CEDAR), University of Cambridge School of Clinical Medicine, Cambridge, UK

**Keywords:** physical activity, policy, longitudinal studies, public health, public health policy

## Abstract

**Background:**

The use of private motor vehicles places a considerable burden on public health. Changes in workplace car parking policies may be effective in shifting behaviour. We use a natural experimental design to assess whether changes in policy were associated with differences in commute mode.

**Methods:**

We used cohort data from participants working in Cambridge (2009–2012). Commuters reported their trips and travel modes to work over the last week, workplace car parking policy and socioeconomic, environmental and health characteristics. Changes in policy were defined between phases (1608 transition periods; 884 participants). Using generalised estimating equations, we estimated associations between changes in parking policy and the proportion of trips that (i) were exclusively by motor vehicle, (ii) involved walking or cycling and (iii) involved public transport at follow-up.

**Results:**

25.1% of trips were made by motor vehicle, 54.6% involved walking or cycling and 11.7% involved public transport. The introduction of free or paid workplace parking was associated with higher proportions of motor vehicle trips (11.4%, 95% CI (6.4 to 16.3)) and lower proportions involving walking or cycling (−13.3%, 95% CI (−20.2 to –6.4)) and public transport (−5.8%, 95% CI (−10.6 to –0.9)) compared with those with no workplace parking. Restrictive changes in policy were associated with shifts in the expected direction but these were not statistically significant.

**Conclusion:**

Relaxation of parking policy was associated with higher proportions of trips made by motor vehicle. Further longitudinal and intervention research is required to assess generalisability of these findings.

## Introduction

The use of motor vehicles places a considerable burden on public health,^1^ increasing cardio-metabolic risk^2^ and contributing to air pollution and road traffic injuries.^3^ These hazards are compounded by the growth of mass motorisation, planning policies that prioritise motor vehicles and a lack of infrastructure for walking and cycling.^1 4^ Evidence suggests that replacing private motor vehicle trips with more active modes of transport is likely to have a positive effect on physical activity and obesity rates^5^ and the risk of numerous non-communicable diseases.^4^ These benefits may outweigh risks from injury or exposure to air pollution.^6^ Accordingly, the shift of travel away from private motor vehicles has become an important goal of public policy.^7–9^

In the UK, the National Institute for Health and Care Excellence has undertaken a series of extensive evidence reviews to identify the strategies most likely to encourage and enable adults to walk and cycle. These range from individual-level behaviour change programmes and group-level incentives, to physical changes in the built environment.^10–12^ Of these approaches, individually delivered strategies, such as the direct provision of advice or information, may prove labour-intensive, while new transport infrastructures may be unaffordable when budgets are limited.^13^ By contrast, policy changes have been implemented successfully as an effective means of moderating negative health behaviours such as smoking^14^ and alcohol consumption.^15^ In the context of active travel, these could include negative incentives that penalise car use or positive incentives that reward active travel. Unfortunately, recent reviews indicate that the quality of existing evidence for the impact of incentives on travel behaviour is poor,^12 16^ with studies focussing on an narrow range of interventions and designs that offer a weak basis for causal inference. Incentives and disincentives for motor vehicle use thus represent an insufficiently researched but potentially promising avenue for encouraging healthier behaviours,^16^ with the possibility that congestion charging schemes may be effective at increasing walking, cycling and public transport use.^12^

It is plausible that similar policies in workplace settings may also encourage shifts away from motor vehicles and towards alternative modes of travel. This view is supported by a study of 20 exemplar cases in the UK, which suggests that parking management (such as the introduction of permits, charges or compensation for not using a private vehicle) was the single most important factor in achieving behaviour change.^17^ The potential public health impact if such schemes were widely adopted may be sizeable, with motor vehicles listed as the usual mode of travel for 64% of commutes in the UK in 2016.^18^ While cross-sectional studies indicate that employees with paid parking or no parking access are more likely to walk, cycle or use public transport,^19^ it is unknown whether changes in policy lead to changes in behaviour. A recent systematic review of workplace interventions to promote active travel^20^ identified only one natural experimental study to have incorporated a change in parking policy.^21^ The authors used data from two repeat cross-sectional travel surveys 6 years apart and found a reduction in the proportion of commutes undertaken by motor vehicle at a workplace where parking charges and access restrictions were implemented alongside incentives for walking and cycling. The reduction in motor vehicle use was substantially greater than reported at a workplace where only incentives for active travel were used. Although the study describes changes in the proportion of staff travelling to work by four transport modes between 2006 and 2012, statistical tests of these changes were only performed for the main outcome of driving to work alone.

Robust evidence about the efficacy of workplace parking policies is lacking. Well-controlled longitudinal studies are required to better establish the suitability of workplace parking policies as a potential avenue for improving public health. For this reason, we used longitudinal data from working-age adults to conduct a quasi-experiment which examined the impact of changes in workplace car parking policy between free, paid and no parking on the proportions of commuting trips that were (i) made exclusively by motor vehicle, (ii) involved walking and/or cycling and (iii) involved public transport (bus and/or train). It was hypothesised that the introduction of more restrictive parking policies would be associated with transitions away from exclusive car use and toward the incorporation of walking, cycling and public transport use.

## Methods

### Study population

We used data from the Commuting and Health in Cambridge study—a prospective cohort of adults who were aged ≥16 years at enrolment, worked in Cambridge, UK, and lived within 30 km of the city. The proportion of employees who travel to work by car is lower in the Greater Cambridge than in the UK as a whole,^22^ with the city itself exhibiting a distinct cycling culture that derives from multiple factors, including flat topography.^23^ Further information concerning study recruitment and data collection is available elsewhere.^23^ A total 1164 employees were enrolled between May and December 2009 (phase 1), with participants re-contacted in 2010 (phase 2), 2011 (phase 3) and 2012 (phase 4). At each phase, participants were asked to complete a postal questionnaire pertaining to their lifestyle, commute, workplace, environment and health. By the end of the study, 1427 employees provided data, of whom 993 participated in at least two consecutive phases. For each consecutive pair, the first phase is hereafter referred to as the ‘baseline’ and the second as the ‘follow-up’.

### Workplace parking policies

Commuters were asked to report whether or not their workplace offered ‘free’, ‘paid’ or ‘no’ workplace parking. As shown in table 1, changes in parking policy between consecutive phases were grouped according to whether they were more or less restrictive of motor vehicle use. Unchanged policies were defined as ‘stable’.

**Table 1:** Proportions of trips by category of workplace parking policy at baseline and follow-up within the complete case sample

Workplace parking policies	Participants (n)	Transition periods (t)
Transitions to less restrictive policies		
Stable no parking (reference)	206	340
No parking to free or paid parking	85	105
Stable paid parking (reference)	233	431
Paid parking to free parking	16	27
Transitions to more restrictive policies		
Stable free parking (reference)	359	603
Free parking to paid or no parking	51	66
Stable paid parking (reference)	232	431
Paid parking to no parking	21	36

### Modes of travel

The modes of transport used on the commute were obtained at each phase from a 7-day diary. Participants were asked to record all the modes used for each trip to and from work. Seven options were provided: ‘bus or coach’, ‘train or underground’, ‘car, taxi or van’, ‘motorcycle or moped’, ‘bicycle’, ‘walking’ and ‘other’. Participants were able to indicate if they had not travelled to work on a given day.

Given that participants reported different numbers of weekly trips (eg, working at home on some days or working part-time), we defined three outcome variables that describe the proportions of trips that (i) were exclusively by motor vehicle (‘car, taxi or van’ or ‘motorcycle or moped’), (ii) involved walking and/or cycling either alone or in combination with other modes (‘bicycle’ and/or ‘walking’) and (iii) involved public transport (‘bus or coach’ and/or ‘train or underground) alone or in combination.

### Covariates

Questionnaire data were used to define age, date of questionnaire completion, highest educational qualification, household car access, commute distance, physical and mental health, perceptions of the commute environment, and attitudes towards, enjoyment of and habits for car use. A full description of these covariates, their derivation and the rationale for their inclusion is provided in the online supplementary appendix 1.

10.1136/jech-2018-210983.supp1 Supplementary file 1

### Statistical analysis

We included data from participants who reported being employed across at least two consecutive phases and had complete data on outcomes, exposures and covariates. Utilising the -xtgee- and -margins- packages in Stata 14,^24^ we used generalised estimating equations (GEEs) to estimate associations between changes in policy and the proportions of commute trips made by different modes of travel at follow-up. All analyses were adjusted for the proportion of trips reported at baseline. This method is equivalent to modelling a change score with adjustment for baseline travel behaviour.^25 26^ An unstructured correlation matrix was specified to allow for the non-independence of data from commuters who participated across multiple consecutive pairs of phases. Because the outcomes were proportions bounded by 0 and 1, the assumption of normally distributed residuals was not appropriate. A binomial distribution with a logit link function was therefore used (ie, a fractional logit GEE model).

Covariates were included as follows: model 1 (baseline proportion of trips); model 2 (model 1, plus baseline age, education and home ownership, and baseline and follow-up season; model 3 (model 2, plus baseline car access, commute distance, perceptions of the environment and Theory of Planned Behaviour variables); model 4 (model 3, plus baseline physical and mental health score and PCS); model 5 (model 4, plus any changes in home or work location).

As we anticipated that the introduction of restrictive parking policies would have a greater impact on commuters who travelled exclusively by motor vehicle, we repeated our analyses restricted to these individuals. Where the number of participants permitted, the changes in workplace parking policy described in table 1 were disaggregated into more detailed categories. These have smaller sub group sample sizes but provide less heterogeneous and more specific comparisons.

### Role of the funding source

The study funders had no role in study design, data collection, data analysis, data interpretation or writing of the report. The corresponding author had full access to all the data in the study and the final responsibility for the decision to submit for publication.

## Results

### Descriptive statistics

**Figure 1:**
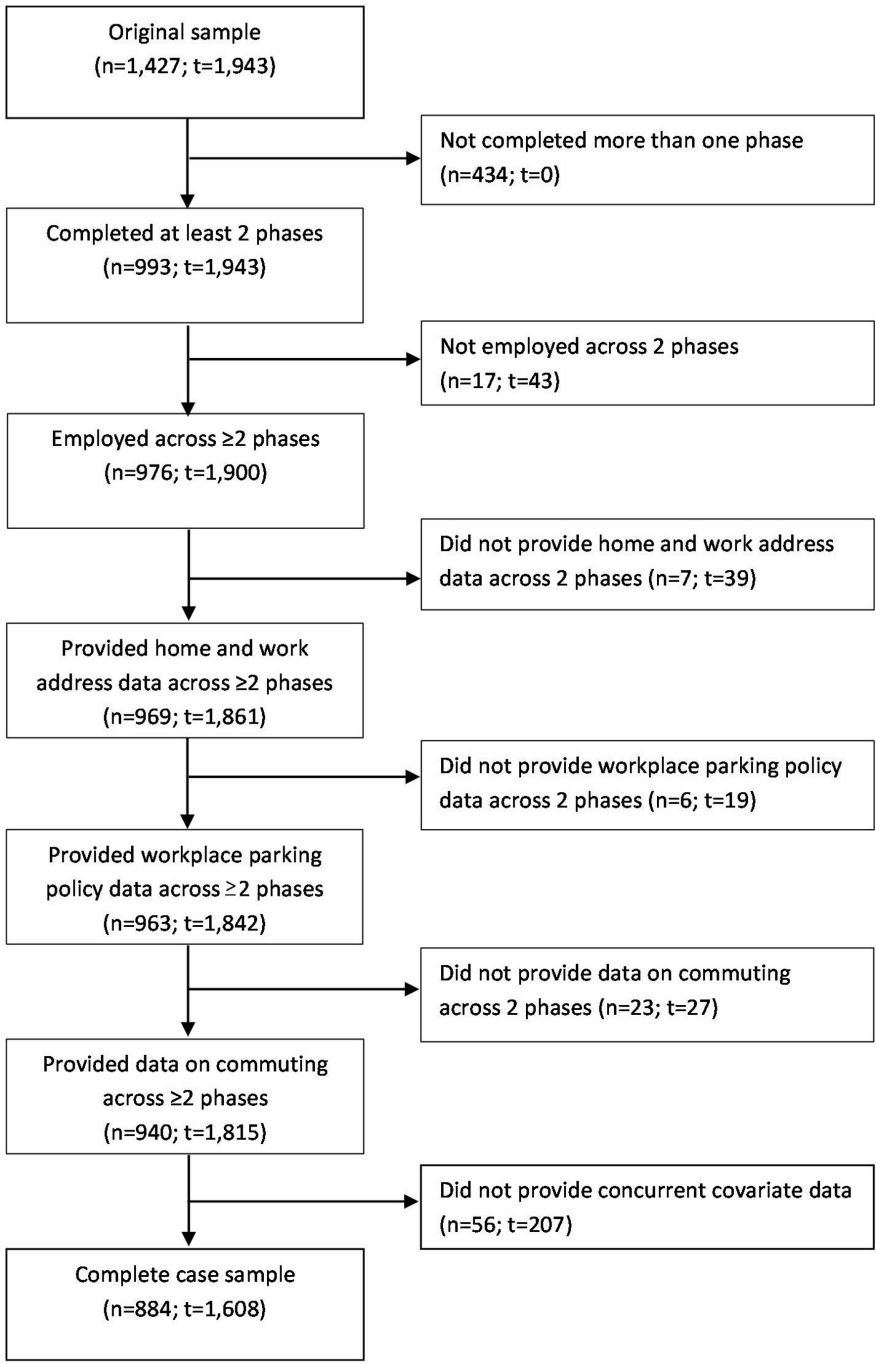
Flow diagram for the derivation of the complete case sample. n, number of participants; t, number of transition periods.

A total 884 participants were employed across ≥2 phases and provided complete data on workplace parking policy, commuting behaviour and covariates (figure 1). These had a mean age of 43.3 (SD 11.1) years, commuted a mean 13.1 (SD 11.4) miles and had access to a mean 1.4 (SD 0.9) cars (online supplementary appendix 2supplementary appendices 2). Of all commute trips reported over a 7-day period, 25.1% were made exclusively by motor vehicle, 54.6% involved an active mode of travel and 11.7% involved public transport. In response to statements concerning perceptions of their route to work, the majority of commuters disagreed or strongly disagreed that their route was ‘pleasant to walk’ and a majority also disagreed or strongly disagreed that their route was ‘safe for cyclists’. Workplace car parking policy remained largely stable, with only 234 (14.6%) of 1608 pairs of observations involving a change of policy.

Online supplementary appendices 3a and 3b show that baseline characteristics at the first phase of observation were broadly comparable among participants within the reference and comparator groups for transitions to less restrictive policies, but more disparate within the equivalent groups for shifts to more restrictive policies. For instance, relative to employees who transitioned from free to paid or no parking, those with stable access to free parking completed a greater number of trips exclusively by motor vehicle, commuted further and had access to a greater number of cars at baseline.

### Transitions to less restrictive workplace parking policies

After adjustment for covariates and baseline differences in the groups, the proportion of trips undertaken exclusively by motor vehicle was (11.4 (95% CI 6.4 to 16.3)%) higher at follow-up among participants who reported that the workplace policy changed from no parking to a free or paid parking, relative to those whose workplaces did not provide car parking (table 2). By contrast, the proportions of trips involving walking and/or cycling (−13.3, 95% CI (−20.2 to –6.4)%) or public transport (−5.8, 95% CI (−10.6 to –0.9)%) were lower. Disaggregated analyses for changes from no to free and no to paid parking are presented in the online supplementary appendix 4. Associations between changes from paid parking to free parking and the proportion of car trips undertaken exclusively by motor vehicle were of similar magnitude but were not statistically significant.

**Table 2:** Transitions to less restrictive workplace parking policies and differences in expected proportions of trips to work by commute mode

Transitions to less restrictive parking policies	Participants (n)	Transition periods (t)	Model 1		Model 2		Model 3		Model 4		Model 5	
			Percentage difference (95% CI)	P values	Percentage difference (95% CI)	P values	Percentage difference (95% CI)	P values	Percentage difference (95% CI)	P values	Percentage difference (95% CI)	P values
Trips exclusively by motor vehicle												
Stable no parking	206	340	Reference		Reference		Reference		Reference		Reference	
No parking to free or paid parking	85	105	15.5 (8.5 to 22.5)	<0.001	15.8 (8.9 to 22.7)	<0.001	12.5 (7.2 to 17.8)	<0.001	13.2 (8.1 to 18.3)	<0.001	11.4 (6.4 to 16.3)	<0.001
Stable paid parking	233	431	Reference		Reference		Reference		Reference		Reference	
Paid parking to free parking	16	27	2.3 (−10.3 to 15.0)	0.717	4.6 (−7.1 to 16.3)	0.442	12.6 (−2.5 to 27.6)	0.102	14.2 (−0.5 to 29.0)	0.059	12.3 (−2.9 to 27.5)	0.114
Trips that involved walking and/or cycling												
Stable no parking	206	340	Reference		Reference		Reference		Reference		Reference	
No parking to free or paid parking	85	105	−18.6 (−25.7 to 11.5)	<0.001	−17.7 (−24.6 to 10.8)	<0.001	−14.7 (−21.4 to 8.0)	<0.001	−15.4 (−22.1 to 8.6)	<0.001	−13.3 (−20.2 to 6.4)	<0.001
Stable paid parking	233	431	Reference		Reference		Reference		Reference		Reference	
Paid parking to free parking	16	27	2.3 (−10.7 to 15.8)	0.702	1.0 (−12.0 to 14.0)	0.881	−11.8 (−26.6 to 3.0)	0.117	−13.0 (-27.6 to 1.6)	0.082	−12.7 (−27.7 to 2.3)	0.096
Trips that involved public transport												
Stable no parking	206	340	Reference		Reference		Reference		Reference		Reference	
No parking to free or paid parking	85	105	−6.8 (−12.0 to 1.5)	0.011	−6.8 (−11.9 to 1.7)	0.009	−6.2 (−11.1 to 1.3)	0.013	−6.4 (−11.3 to 1.6)	0.009	−5.8 (−10.6 to 0.9)	0.020
Stable paid parking	233	431	Reference		Reference		Reference		Reference		Reference	
Paid parking to free parking	16	27	−8.3 (−15.4 to 1.2)	0.021	−8.5 (−16.8 to 0.2)	0.044	−7.4 (−16.4 to 1.7)	0.112	−7.2 (−15.7 to 1.4)	0.102	−6.3 (−14.6 to 2.1)	0.141

Model 1: adjusted for the proportion of trips before transition by each respective commute mode.

Model 2: as model 1, plus age (before transition), season (before and after transition), education (before transition) and home ownership (before transition).

Model 3: as model 2, plus car access (before transition), commute distance (before transition), Theory of Planned Behaviour (before transition) and perceptions of the environment (before transition).

Model 4: as model 3, plus mental and physical health component scores (before transition).

Model 5: as model 4, plus change to work address and change to home address.

### Transitions to more restrictive workplace parking policies

For those participants who reported changes from free parking to paid or no parking, small but non-significant differences in commute trips were evident, including those trips completed exclusively by motor vehicle (table 3: −3.7, 95% CI (−10.8 to 3.5)%), or that involved either walking and/or cycling (0.6, 95% CI (−7.4 to 8.6)%) or public transport (−0.2, 95% CI (−4.9 to 4.5)%), relative to a stable free parking policy. Findings were similar for transitions from paid to no parking. Results from analyses with disaggregated changes in parking policy are reported in the online supplementary appendix 5.

**Table 3:** Transitions to more restrictive workplace parking policies and differences in expected proportions of trips to work by commute mode

Transitions to more restrictive parking policies	Participants (n)	Transition periods (t)	Model 1		Model 2		Model 3		Model 4		Model 5	
			Percentage difference (95% CI)	P values	Percentage difference (95% CI)	P values	Percentage difference (95% CI)	P values	Percentage difference (95% CI)	P values	Percentage difference (95% CI)	P values
Trips exclusively by motor vehicle												
Stable free parking	359	603	Reference		Reference		Reference		Reference		Reference	
Free parking to paid or no parking	51	66	−8.0 (−16.5 to 0.4)	0.062	−7.9 (−16.4 to 0.5)	0.065	−5.0 (−11.9 to 1.8)	0.151	−4.3 (−11.2 to 2.7)	0.228	−3.7 (−10.8 to 3.5)	0.316
Stable paid parking	232	431	Reference		Reference		Reference		Reference		Reference	
Paid parking to no parking	21	36	−6.7 (−17.4 to 3.9)	0.219	−7.6 (−18.1 to 2.9)	0.157	−2.0 (−15.3 to 11.3)	0.769	−2.3 (−14.7 to 10.1)	0.711	−2.5 (−15.1 to 10.1)	0.697
Trips that involved walking and/or cycling												
Stable free parking	359	603	Reference		Reference		Reference		Reference		Reference	
Free parking to paid or no parking	51	66	8.2 (−1.9 to 18.3)	0.11	9.4 (−0.9 to 19.7)	0.074	1.6 (−6.6 to 10.0)	0.695	1.3 (−6.9 to 9.4)	0.76	0.6 (−7.4 to 8.6)	0.878
Stable paid parking	232	431	Reference		Reference		Reference		Reference		Reference	
Paid parking to no parking	21	36	6.1 (−6.3 to 18.6)	0.336	6.6 (−7.1 to 20.4)	0.344	0.7 (−12.2 to 13.7)	0.912	0.5 (−12.3 to 13.2)	0.942	0.3 (−12.4 to 13.0)	0.965
Trips that involved public transport												
Stable free parking	359	603	Reference		Reference		Reference		Reference		Reference	
Free parking to paid or no parking	51	66	−7.8 (−5.7 to 4.2)	0.760	−0.9 (−5.5 to 3.8)	0.716	−0.2 (−4.7 to 4.4)	0.947	−0.2 (−4.8 to 4.4)	0.940	−0.2 (−4.9 to 4.5)	0.925
Stable paid parking	232	431	Reference		Reference		Reference		Reference		Reference	
Paid parking to no parking	21	36	0.4 (−12.6 to 13.3)	0.956	0.1 (−11.4 to 13.4)	0.878	−0.4 (−12.3 to 11.5)	0.948	−0.6 (−12.4 to 11.3)	0.927	0.7 (−11.2 to 12.5)	0.911

Model 1: adjusted for the proportion of trips before transition by each respective commute mode.

Model 2: as model 1, plus age (before transition), season (before and after transition), education (before transition) and home ownership (before transition).

Model 3: as model 2, plus car access (before transition), commute distance (before transition), Theory of Planned Behaviour (before transition) and perceptions of the environment (before transition).

Model 4: as model 3, plus mental and physical health component scores (before transition).

Model 5: as model 4, plus change to work address and change to home address.

### Transitions among exclusive motor vehicle users

We intended to report multivariable-adjusted models restricted to participants who travelled exclusively by motor vehicle on at least one occasion in the last week. Unfortunately, owing to small cell sizes, a number of models would not converge even when included covariates were substantially reduced in number. These models are therefore not reported.

## Discussion

This is the one of the first natural experimental studies to use cohort data to assess the role that changes in workplace parking policies may play in changing commuting patterns. Relaxations of parking policies (ie, changes that were less restrictive) were associated with higher proportions of commute trips made exclusively by motor vehicle and, in line with a causal interpretation and substitution, we found corresponding lower proportions of trips involving walking, cycling or public transport. Reverse associations were evident following the introduction of more restrictive workplace parking policies. Although consistent with our hypothesis, these latter associations were small in magnitude and not statistically significant. It is plausible that stronger associations may be observed in larger cohorts where greater numbers of people experience changes in workplace parking policies or where baseline levels of car use are higher. The use of private motor vehicles is low in Cambridge, relative to the UK as a whole, and it may be that Cambridge commuters who use motor vehicles are particularly dependent on them or else less able or willing to stop using them. As such, our study in this cohort may have had less ability to detect favourable differences in motor vehicle use following the introduction of restrictive car parking policies. Nevertheless, given the high prevalence of car use in many countries, even modest reductions in motor vehicle trips may still elicit sizeable benefits for public and environmental health at the population level. For example, a 3.7% decline could equate to a decrease of around 7 31 136 commutes by car across the UK (given a mid-year UK population estimate of 41 443 872 in 2016 for persons aged 16–64 years,^27^ a mid-year employment rate of 74.5%,^27^ and an estimated 64% of commute trips involving travel exclusively by car or van^18^). Importantly, reductions are most likely to be concentrated among those with shorter commutes,^19^ helping to target congestion and air pollution in urban environments.

Our findings are in line with those from cross-sectional studies, which indicate that commuters with access to free or paid car parking at work are less likely to walk or cycle as part of the journey to work.^19^ However, our results show associations of smaller magnitude than reported in previous prospective studies.^21 28^ One possible reason for this may be these studies were not adjusted for individual characteristics and other factors that may influence travel behaviour.^20^ Alternatively, smaller associations could be indicative of our study’s singular focus on disincentives for motor vehicle travel. It is plausible that incentives may be required alongside restrictive parking policies if larger shifts toward more active modes of commuting are to be realised. This strategy is being adopted in the UK^29^ and matches the approach proposed by governmental and intergovernmental actors.^7–9^

Despite agreement that ‘carrots’ and ‘sticks’ are likely to be important for the promotion of active commuting, there appears to be little clarity about which components or combination of components are most effective. Where implemented,^21 29^ interventions have combined multiple elements in a fashion that complicates any conclusion as to their relative efficacy. To provide a stronger evidence base for policy and practice, studies of comparative effectiveness are required. Such research should pay particular attention to effects within population subgroups, such as those on lower incomes or with longer commutes, to identify any potentially negative implications for health equity.^30 31^

A further issue concerns the feasibility of encouraging employers to implement changes and be agents of change. Although resistance from employees is common, there is evidence that successful implementation can be encouraged through incentives for those who do not drive to work, and a raising of awareness that parking is a limited and costly resource.^17 21^ Further research concerning acceptability and identifying barriers and enablers to successful introduction is required.

### Strengths and limitations

Our study has a number of strengths. First, by examining multivariable-adjusted associations between shifts in policy and travel behaviour, we provide a better indication than current evidence as to the impact of policy changes at the population level, and provide a stronger basis for causal inference than other methods.^32^ Although our natural experiment lacked randomisation by design, we adjusted for observed differences between groups and accounted for confounding from unobserved time-invariant factors. Additionally, we adjusted for the baseline proportion of trips by commute mode, accounting for differences present at the outset and helping to rule out the effect of regression to the mean.^25^ Collectively, this analytical approach and the use of multiple comparison groups is recommended in current guidance on natural experimental studies.^32^ Adjusted models show consistent associations in the expected directions across multiple comparison groups, particularly for less restrictive policies, further strengthening the case for causal inference.

We used detailed data on commuting and captured all modes used within a trip. This is in contrast to most previous studies, where the main or usual mode of travel to work has been used,^19^ or where the impact on car trips has been assessed without considering the effect on alternative modes of travel.^21 28^ Our approach specifically allowed us to identify small but otherwise overlooked changes in travel behaviour, such as the integration of walking or cycling alongside a longer car journey. Such transitions in commute behaviour may be more realistic and practical than full modal shifts away from private motor vehicles, particularly for those living in rural areas.

Some limitations remain. First, as changes in workplace parking policies and travel behaviour were assessed concurrently between phases, reverse causation is possible. For this same reason, it was not appropriate to adjust for time-varying factors. Associations may therefore be attributable to confounding by time-varying factors, such as shifts in the local transport infrastructure, non-workplace parking, and the social acceptability of walking and cycling. The relatively small proportion of participants who reported changes in parking policy limited the depth of analysis, ruling out restricted models and interactions between changes in policy and variables such as commute distance. These more detailed analyses would have helped to elucidate on factors that may modify the relationship between shifts in workplace parking policy and transport choice. Although multiple imputation was considered to account for item non-response, the increase in sample size would have been negligible (around 4%). Our findings may not be generalisable to different geographic areas and populations.

## Conclusion

To our knowledge, this is the first cohort study to have explored the impact of changes in workplace car parking policy on travel behaviour. Our findings indicate that a relaxation of parking policies was associated with higher proportions of trips made exclusively by motor vehicle, and lower proportions of trips involving walking, cycling and public transport, compared with those who reported consistently restrictive parking policies. Transitions to more restrictive policies indicate shifts in travel behaviour in the expected direction. Workplace parking policies may have a sizeable population-level impact on active commuting, particularly in urban areas. Further longitudinal and interventional studies are required to establish the generalisability of these findings and the comparative effectiveness of different incentives for active commuting.

**What is already known on this subject**

- Cross-sectional evidence suggests that employees who pay for parking or have no parking access are more likely to report walking, cycling and public transport use, and are less likely to use a car than workers with free parking. Longitudinal evidence is sparse and a recent systematic review of intervention studies identified only one pre–post natural experimental study, which found reductions in the proportion of commute trips by motor vehicle after incentives for active travel were combined with parking charges and access restrictions.

**What this study adds**

- This study adopts a longitudinal, quasi-experimental approach to elucidate the impact of changes in workplace parking policies on the proportion of trips (i) undertaken exclusively by motor vehicle, (ii) involving walking or cycling and (iii) involving public transport.
- The study indicates that relaxation of parking policies was associated with proportions of exclusive motor vehicle trips that were >10% higher than at workplaces where more restrictive parking policies were consistently in force. By contrast, the introduction of more restrictive policies was associated with small and non-significant differences in travel patterns at follow-up, including higher proportions of trips involving walking and/or cycling. It is possible that larger associations may be achievable through the implementation of policies that both discourage the use of motor vehicles and incentivise more active modes of travel. At the population level, even these small effects may still have a sizeable impact on the number of commutes undertaken by motor vehicle, particularly in urban areas. Further longitudinal and interventional studies are required to establish the generalisability of these findings.
