# Supplementary material for: Changes in workplace car parking and commute mode: a natural experimental study
Source: J Epidemiol Community Health. 2018 Oct 3;73(1):42–9. doi: 10.1136/jech-2018-210983 (PMC6446994; doi:10.1136/jech-2018-210983)
Supplement: Supplementary file 1 [file jech-2018-210983supp001.pdf]

Appendix 1 Derivation of substantive variables

Demographic variables

Age (years) is defined according to the value reported before transition. Highest educational qualification (Degree, NVQ4, NVQ5 or equivalent; BTEC, BEC, TEC (Higher); GCE 'A' Level, Scottish Higher, BTEC, TEC, BEC (National); GCSE Grade A-C, GCSE 'O' Level; other or no formal qualifications) and home ownership (rent or shared ownership; own or other) before transition are also included, as well as the season during which each questionnaire was completed (spring; summer; autumn; winter). Home ownership and education are each correlated in this dataset with the choice of commute mode,[1] while commuters may be more resistant to shifting away from car use if a parking policy transition occurred during colder months.

Car access and commute characteristics

Commute distance (miles) before transition is included as a means of capturing the possibility that participants with longer commute distances will be more resilient to changes in workplace parking policy, with shorter commutes correlated with active modes of travel.[2] Distance was split into tertiles in an effort to capture possible non-linear associations between distance and mode of travel.

Household car access before transition was defined as the total number of cars or vans owned or available for use by members of the participant's household, with car access being correlated with less active commuting.[2]

Theory of Planned Behaviour

The study also includes adjustment for variables designed around the Theory of Planned Behaviour (TPB), which in this context specifies links between people's beliefs and their behaviour toward car use.[3] The theory comprises four main components: attitudes (i.e. the extent to which car use is perceived to be good, beneficial or enjoyable); subjective norms (i.e. perceived pressure from important others to use a car); behavioural control (i.e. perceived barriers and facilitators of car use); and intent (i.e. inclination toward using a car). In addition to explaining variance in physical activity,[4] these components are predictors of active commuting[1,2]

Each of the four components was assessed using eight statements, as outlined in Box 3.[2] Participants were permitted to answer each statement using one of five Likert responses (strongly agree; agree; neither agree nor disagree; disagree; strongly disagree). In each case, values reported before transition were used.

Because of the limited number of transition periods captured within some categories of the Theory of Planned Behaviour variables, these variables were treated as continuous (i.e. assuming a linear association across categories).

Theory of Planned Behaviour statements

Attitudes
'For me to get to and from work next time it would be good to use a car.'
'For me to get to and from work next time it would be pleasant to use a car.'

Subjective norms
'For me to get to and from work next time, most people who are important to me would support my using a car.'
'For me to get to and from work next time, most people who are important to me think I should use a car.'

Behavioural control
'For me to get to and from work next time, it would be easy for me to use a car.'
'For me to get to and from work next time, I would be able to use a car.'

Intent
'For me to get to and from work next time, I intend to use a car.'
'For me to get to and from work next time, I am likely to use a car.'

Mental and physical health

Mental and physical health was assessed using the SF-8, a validated quality of life instrument that comprises single-item scales covering eight domains of general health.[5] Mental and physical health was accounted for using continuous component scores derived from the mental (MCS-8) and physical (PCS-8) health domains.

Perceptions of the commute environment

Perceptions of the environment between home and work have been associated with active commuting.[2,6] To better isolate the independent effect of workplace parking policies on commute behaviour, seven variables are included that correspond to participant agreement with perceptual statements concerning the commute environment (Box 4).[2] Participants could rate each statement according to one of five Likert responses (strongly agree; agree; neither agree nor disagree; disagree; strongly disagree). In each case, values reported before transition were used.

Because of the limited number of transition periods captured within some categories of the perceived environment variables, these variables were treated as continuous (i.e. assuming a linear association across categories).

Perceived commute environment statements
'It is pleasant to walk.'
'The roads are safe for cyclists.'
'There are convenient routes for walking.'
'There is convenient public transport.'
'There are convenient routes for cycling.'
'There is little traffic.'
'It is safe to cross the road.'

Change of home and work address

Given the possibility that any transition in the mode of travel may result not from a shift in workplace parking policy directly, but a change of work or home location, two binary variables were derived to indicate whether or not there was a change of work or home postcode during the transition period.

Appendix 2 Characteristics of the complete case sample at the first phase of observation

Characteristics at the first phase of observation	% (95% CI)	n
**Exposure and outcome characteristics**		
**Workplace parking policy**		
Free	41.5	387
Paid	28.8	255
None	29.6	262
**Proportion of trips exclusively by motor vehicle**		
Mean and standard deviation	25.1 (37.4)	884
**Proportion of trips involving an active mode of travel**		
Mean and standard deviation	54.6 (42.4)	884
**Proportion of trips involving public transport**		
Mean and standard deviation	11.7 (27.1)	884
**Demographic characteristics**		
**Age (years)**	43.3 (11.1)	884
Mean and standard deviation		
**Education**		
Degree, NVQ4, NVQ5 or equivalent	70.8 (67.7, 73.7)	626
BTEC, BEC, TEC (Higher)	7.9 (6.3, 9.9)	70
GCE ‘A’ Level, Scottish Higher, BTEC, TEC, BEC (National)	8.8 (7.1, 10.9)	78
GCSE Grade A-C, GCSE ‘O’ Level	6.2 (4.8, 8.0)	55
Other or no qualification	6.2 (4.8, 8.0)	55
**Home ownership**		
Rent or shared ownership	23.1 (20.4, 26.0)	204
Own or other	76.9 (74.0, 79.6)	680
**Season**		
Spring	24.0 (21.3, 26.9)	212
Summer	25.2 (22.5, 28.2)	223
Autumn	21.9 (19.3, 24.8)	194
Winter	28.8 (25.9, 31.9)	255
**Car access and commute characteristics**		
**Car access (number of cars or vans owned or available for use)**		
Mean and standard deviation	1.39 (0.87)	884
**Commute distance (miles)**		
Mean and standard deviation	13.1 (11.41)	884

Theory of Planned Behaviour

Attitudes

**'For me to get to and from work next time it would be good to use a car.'**		
Strongly agree	37.6 (34.4, 40.8)	332
Agree	22.9 (20.2, 25.7)	202
Neither agree nor disagree	10.4 (8.6, 12.6)	92
Disagree	13.6 (11.5, 16.0)	120
Strongly disagree	15.6 (13.4, 18.2)	138
**'For me to get to and from work next time it would be pleasant to use a car.'**		
Strongly agree	28.2 (25.3, 31.2)	249
Agree	25.0 (22.3, 28.0)	221
Neither agree nor disagree	21.4 (18.8, 24.2)	189
Disagree	16.4 (14.1, 19.0)	145
Strongly disagree	9.0 (7.3, 11.1)	80

Subjective norms

**'For me to get to and from work next time, most people who are important to me would support my using a car.'**		
Strongly agree	29.3 (26.4, 32.4)	259
Agree	24.0 (21.3, 26.9)	212
Neither agree nor disagree	20.8 (18.3, 23.6)	184
Disagree	13.1 (11.0, 15.5)	116
Strongly disagree	12.8 (10.7, 15.2)	113
**'For me to get to and from work next time, most people who are important to me think I should use a car.'**		
Strongly agree	22.4 (19.8, 25.3)	198
Agree	17.3 (14.9, 20.0)	153
Neither agree nor disagree	6.3 (4.9, 8.1)	56
Disagree	29.2 (26.3, 32.3)	258
Strongly disagree	24.8 (22.0, 27.7)	219

Behavioural control

**'For me to get to and from work next time, it would be easy for me to use a car.'**	14.8 (12.6, 17.3)	131
Strongly agree	9.8 (8.0, 12.0)	87
Agree	5.3 (4.0, 7.0)	47
Neither agree nor disagree	37.7 (34.5, 40.9)	333
Disagree	32.4 (29.3, 35.5)	286
Strongly disagree		
**'For me to get to and from work next time, I would be able to use a car.'**		

Strongly agree	45.1 (41.9, 48.4)	399
Agree	14.6 (12.4, 17.1)	129
Neither agree nor disagree	5.0 (3.7, 6.6)	44
Disagree	14.7 (12.5, 17.2)	130
Strongly disagree	20.6 (18.0, 23.4)	182
<u>Intent</u>		
**'For me to get to and from work next time, I intend to use a car.'**	42.8 (39.5, 46.1)	378
Strongly agree	16.1 (13.8, 18.6)	142
Agree	3.7 (2.7, 5.2)	33
Neither agree nor disagree	13.7 (11.6, 16.1)	121
Disagree	23.8 (21.1, 26.7)	210
Strongly disagree		
**'For me to get to and from work next time, I am likely to use a car.'**		
Strongly agree	9.6 (7.8, 11.7)	85
Agree	11.9 (9.9, 14.2)	105
Neither agree nor disagree	23.9 (21.2, 26.8)	211
Disagree	33.6 (30.6, 36.8)	297
Strongly disagree	21.0 (18.5, 23.9)	186
**<u>Perceptions of the commute environment</u>**		
**‘It is pleasant to walk.’**	3.6 (2.6, 5.1)	32
Strongly agree	21.4 (18.8, 24.2)	189
Agree	17.3 (14.9, 20.0)	153
Neither agree nor disagree	42.3 (39.1, 45.6)	374
Disagree	15.4 (13.1, 17.9)	136
Strongly disagree	22.4 (19.8, 25.3)	198
	17.3 (14.9, 20.0)	153
**‘The roads are safe for cyclists.’**	6.3 (4.9, 8.1)	56
Strongly agree	29.2 (26.3, 32.3)	258
Agree	24.8 (22.0, 27.7)	219
Neither agree nor disagree		
Disagree		
Strongly disagree	14.8 (12.6, 17.3)	131
**‘There are convenient routes for walking.’**		
Strongly agree	16.9 (14.5, 19.5)	149
Agree	38.5 (35.3, 41.7)	340
Neither agree nor disagree	20.1 (17.6, 22.9)	178
Disagree	12.7 (10.6, 15.0)	112
Strongly disagree	11.9 (9.9, 14.2)	105
**‘There is convenient public transport.’**		

Strongly agree	21.8 (19.2, 24.7)	193
Agree	29.2 (26.3, 32.3)	258
Neither agree nor disagree	14.3 (12.1, 16.7)	126
Disagree	27.8 (25.0, 30.9)	246
Strongly disagree	6.9 (5.4, 8.8)	61
**'There are convenient routes for cycling.'**		
Strongly agree	10.6 (8.8, 12.8)	94
Agree	17.3 (14.9, 20.0)	363
Neither agree nor disagree	16.6 (14.3, 19.2)	147
Disagree	41.1 (37.9, 44.3)	153
Strongly disagree	14.4 (12.2, 16.8)	94
**'There is little traffic.'**		
Strongly agree	46.3 (43.0, 49.6)	409
Agree	36.5 (33.4, 39.8)	323
Neither agree nor disagree	7.2 (5.7, 9.1)	64
Disagree	6.9 (5.4, 8.8)	61
Strongly disagree	3.1 (2.1, 4.4)	27
**'It is safe to cross the road.'**		
Strongly agree	7.4 (5.8, 9.3)	65
Agree	13.7 (11.6, 16.1)	121
Neither agree nor disagree	27.9 (25.1, 31.0)	247
Disagree	43.7 (40.4, 47.0)	386
Strongly disagree	7.4 (5.8, 9.3)	65
**Health**		
**MCS-8[a]**		
Score (mean)	51.13 (7.77)	884
**PCS-8[b]**		
Score (mean)	54.00 (5.98)	884

[a]Mental component score from the mental health domain of the SF-8 quality-of-life instrument; [b]Physical component score from the mental health domain of the SF-8 quality-of-life instrument

Appendix 3a Characteristics of the complete case sample at the first phase of observation, by transition to less restrictive parking policies

	Transitions to less restrictive parking policies					
	No parking to free or paid parking (n=85; t=105)	Stable no parking (n=206; t=340)		Paid parking to free parking (n=16; t=27)	Stable paid parking (n=233; t=431)	
**Characteristics at the first phase of observation**	**% (95% CI)**	**% (95% CI)**	**p for difference**	**% (95% CI)**	**% (95% CI)**	**p for difference**
**Outcome characteristics**						
**Proportion of trips exclusively by motor vehicle**						
Mean and standard deviation	10.2 (26.4)	6.4 (18.1)	0.300	25.6 (39.6)	26.9 (38.7)	0.906
**Proportion of trips involving an active mode of travel**						
Mean and standard deviation	67.8 (41.0)	65.9 (37.3)	0.764	47.3 (44.8)	51.7 (42.4)	0.730
**Proportion of trips involving public transport**						
Mean and standard deviation	13.0 (27.9)	15.3 (30.8)	0.594	7.3 (21.1)	10.5 (25.4)	0.603
**Demographic characteristics**						
**Age (years)**						
Mean and standard deviation	40.6 (11.0)	43.7 (11.1)	0.056	33.6 (8.5)	42.4 (10.5)	<0.001
**Education**						
Degree, NVQ4, NVQ5 or equivalent	67.8 (54.5, 78.7)	74.4 (67.4, 80.4)	0.383	69.2 (36.5, 89.8)	70.2 (63.6, 76.1)	0.245
BTEC, BEC, TEC (Higher)	8.5 (3.5, 19.2)	5.7 (3.1, 10.3)		7.7 (0.8, 46.9)	5.9 (3.3, 10.1)	
GCE ‘A’ Level, Scottish Higher, BTEC, TEC, BEC (National)	13.6 (6.8, 25.3)	6.3 (3.5, 11.0)		23.1 (6.3, 57.2)	8.8 (5.6, 13.6)	
GCSE Grade A-C, GCSE ‘O’ Level	5.1 (1.6, 15.1)	6.3 (3.5, 11.0)		-	7.3 (4.4, 11.8)	
Other or no qualification	5.1 (1.6, 15.1)	7.4 (4.3, 12.4)		-	7.8 (4.8, 12.4)	
**Car access and commute characteristics**						
**Car access (number of cars or vans owned or available for use)**						
Mean and standard deviation	1.4 (1.1)	1.1 (0.8)	0.090	1.3 (0.9)	1.6 (0.9)	0.267
**Commute distance (miles)**						
Mean and standard deviation	10.9 (10.7)	9.5 (9.9)	0.385	14.2 (14.6)	15.3 (11.5)	0.802

Appendix 3b Characteristics of the complete case sample at the first phase of observation, by transition to more restrictive parking policies

	Transitions to more restrictive parking policies					
	**Free parking to paid or no parking (n=51; t=66)**	**Stable free parking (n=359; t=603)**		**Paid parking to no parking (n=31; t=36)**	**Stable paid parking (n=232; t=431)**	
**Characteristics at the first phase of observation**	**% (95% CI)**	**% (95% CI)**	**p for difference**	**% (95% CI)**	**% (95% CI)**	**p for difference**
**Outcome characteristics**						
**Proportion of trips exclusively by motor vehicle**						
Mean and standard deviation	16.6 (27.6)	39.4 (41.7)	<0.001	7.1 (24.4)	26.9 (38.7)	0.003
**Proportion of trips involving an active mode of travel**						
Mean and standard deviation	62.0 (39.9)	43.4 (42.6)	0.011	86.8 (30.0)	51.7 (42.4)	<0.001
**Proportion of trips involving public transport**						
Mean and standard deviation	19.9 (35.5)	8.0 (23.1)	0.059	5.9 (18.8)	10.5 (25.4)	0.348
**Demographic characteristics**						
**Age (years)**						
Mean and standard deviation	40.4 (12.4)	43.8 (10.7)	0.121	39.9 (13.7)	42.4 (10.5)	0.459
**Education**						
Degree, NVQ4, NVQ5 or equivalent	70.6 (52.5, 83.9)	70.5 (65.1, 75.4)	0.720	58.8 (32.7, 80.7)	70.2 (63.6, 76.1)	0.242
BTEC, BEC, TEC (Higher)	11.8 (4.3, 28.6)	10.5 (7.5, 14.5)		5.9 (0.7, 37.3)	5.9 (3.3, 10.1)	
GCE ‘A’ Level, Scottish Higher, BTEC, TEC, BEC (National)	11.8 (4.3, 28.6)	8.5 (5.9, 12.3)		23.5 (8.1, 51.8)	8.8 (5.6, 13.6)	
GCSE Grade A-C, GCSE ‘O’ Level	5.9 (1.4, 22.0)	5.6 (3.5, 8.8)		11.8 (2.5, 40.9)	7.3 (4.4, 11.8)	
Other or no qualification	-	4.9 (3.0, 8.0)		-	7.8 (4.8, 12.4)	
**Car access and commute characteristics**						
**Car access (number of cars or vans owned or available for use)**						
Mean and standard deviation	1.1 (0.9)	1.4 (0.8)	0.048	1.3 (0.7)	1.6 (0.9)	0.108
**Commute distance (miles)**						
Mean and standard deviation	9.4 (11.5)	14.1 (11.1)	0.024	10.9 (13.9)	15.3 (11.5)	0.208

Appendix 4 A detailed breakdown of transitions to less restrictive workplace parking policies and differences in expected proportions of trips to work by commute mode

			Model 1		Model 2		Model 3		Model 4		Model 5	
**Transitions to less restrictive parking policies**	**Participants (n)**	**Transition periods (t)**	**Percentage difference (95% CI)**	**p-value**	**Percentage difference (95% CI)**	**p-value**	**Percentage difference (95% CI)**	**p-value**	**Percentage difference (95% CI)**	**p-value**	**Percentage difference (95% CI)**	**p-value**
**Trips exclusively by motor vehicle**												
Reference: Stable no parking	206	340										
No parking to free parking	59	73	19.1 (10.1, 28.1)	<0.001	17.1 (8.8, 25.3)	<0.001	15.6 (8.8, 22.4)	<0.001	16.2 (9.5, 22.9)	<0.001	13.5 (7.1, 19.8)	<0.001
Reference: Stable no parking	209	340										
No parking to paid parking	26	32	7.1 (-1.2, 15.3)	0.093	8.2 (-0.1, 16.6)	0.053	5.4 (-2.1, 12.9)	0.156	5.7 (-1.6, 13.0)	0.111	5.3 (-1.9, 12.6)	0.150
**Trips that involved walking and/or cycling**												
Reference: Stable no parking	206	340										
No parking to free parking	59	73	-19.5 (-28.1, -11.0)	<0.001	-18.8 (-27.3, 10.3)	<0.001	-16.4 (-24.7, -8.2)	<0.001	-16.9 (-25.3, -8.4)	<0.001	-13.6 (-21.6, -5.7)	0.001
Reference: Stable no parking	209	340										
No parking to paid parking	26	32	-16.5 (-28.5, -4.4)	0.007	-15.8 (-26.8, -4.9)	0.005	-13.3 (-24.5, -2.1)	0.020	-13.6 (-24.4, -2.9)	0.013	-12.8 (-24.2, -1.4)	0.028
**Trips that involved public transport**												
Reference: Stable no parking	206	340										
No parking to free parking	59	73	-8.7 (-14.9, -2.5)	0.006	-8.1 (-14.4, -1.8)	0.011	-7.7 (-13.8, -1.5)	0.014	-8.1 (-14.1, -2.1)	0.008	-6.5 (-12.4, -0.6)	0.032
Reference: Stable no parking	209	340										
No parking to paid parking	26	32	-2.8 (-8.0, 2.4)	0.299	-3.8 (-9.0, 1.4)	0.148	-4.5 (-9.7, 0.8)	0.096	-4.5 (-9.7, 0.8)	0.094	-4.3 (-9.4, 0.8)	0.102

Appendix 5 A detailed breakdown of transitions to more restrictive workplace parking policies and differences in expected proportions of trips to work by commute mode

			Model 1		Model 2		Model 3		Model 4		Model 5	
**Transitions to more restrictive parking policies**	**Participants (n)**	**Transition periods (t)**	**Percentage difference (95% CI)**	**p-value**	**Percentage difference (95% CI)**	**p-value**	**Percentage difference (95% CI)**	**p-value**	**Percentage difference (95% CI)**	**p-value**	**Percentage difference (95% CI)**	**p-value**
**Trips exclusively by motor vehicle**												
Stable free parking	359	603	Reference		Reference		Reference		Reference		Reference	
Free parking to paid parking	12	16	-10.1 (-23.3, 3.0)	0.131	-8.0 (-19.8, 3.9)	0.188	-5.7 (-17.1, 5.6)	0.32	-6.2 (-17.6, 5.2)	0.284	-6.0 (-17.1, 5.2)	0.309
Stable free parking	359	603	Reference		Reference		Reference		Reference		Reference	
Free parking to no parking	39	50	-6.1 (-15.7, 3.6)	0.219	-7.2 (-17.8, 3.4)	0.183	-3.9 (-11.8, 4.1)	0.341	-2.6 (-10.7, 5.6)	0.538	-2.3 (-10.7, 6.1)	0.595
**Trips that involved walking and/or cycling**												
Stable free parking	359	603	Reference		Reference		Reference		Reference		Reference	
Free parking to paid parking	12	16	8.3 (-0.9, 17.6)	0.078	8.9 (-2.8, 20.1)	0.135	0.6 (-14.8, 16.1)	0.938	0.6 (-15.1, 16.2)	0.945	0.2 (-15.2, 15.6)	0.981
Stable free parking	359	603	Reference		Reference		Reference		Reference		Reference	
Free parking to no parking	39	50	8.2 (-5.5, 22.0)	0.241	9.6 (-3.6, 22.9)	0.153	1.6 (-7.6, 10.9)	0.732	0.9 (-8.1, 10.0)	0.838	0.5 (-8.5, 9.6)	0.907
**Trips that involved public transport**												
Stable free parking	359	603	Reference		Reference		Reference		Reference		Reference	
Free parking to paid parking	12	16	0.6 (-9.5, 10.7)	0.913	0.9 (-9.1, 10.9)	0.862	5.4 (-8.4, 19.2)	0.442	5.5 (-7.6, 18.6)	0.407	6.5 (-8.1, 21.2)	0.380
Stable free parking	359	603	Reference		Reference		Reference		Reference		Reference	
Free parking to no parking	39	50	-1.3 (-6.6, 4.1)	0.638	-1.3 (-6.2, 3.7)	0.619	-0.9 (-5.3, 3.5)	0.689	-0.9 (-5.4, 3.4)	0.662	-0.9 (-5.4, 3.7)	0.711

Model 1: Adjusted for the proportion of trips before transition by each respective commute mode.
Model 2: As Model 1, plus age (before transition), season (before and after transition), education (before transition) and home ownership (before transition).
Model 3: As Model 2, plus car access (before transition), commute distance (before transition), Theory of Planned Behaviour (before transition) and perceptions of the environment (before transition).
Model 4: As Model 3, plus mental and physical health component scores (before transition).
Model 5: As Model 4, plus change to work address and change to home address.

References

1. Panter J, Griffin S, Dalton AM, Ogilvie D. Patterns and predictors of changes in active commuting over 12months. Prev Med. 2013 Dec 1;57(6):776–84.

2. Panter J, Griffin S, Jones A, Mackett R, Ogilvie D. Correlates of time spent walking and cycling to and from work: baseline results from the commuting and health in Cambridge study. Int J Behav Nutr Phys Act. 2011 Nov 10;8:124.

3. Hardeman W, Sutton S, Griffin S, Johnston M, White A, Wareham NJ, et al. A causal modelling approach to the development of theory-based behaviour change programmes for trial evaluation. Health Educ Res. 2005 Dec;20(6):676–87.

4. French DP, Sutton S, Hennings SJ, Mitchell J, Wareham NJ, Griffin S, et al. The Importance of Affective Beliefs and Attitudes in the Theory of Planned Behavior: Predicting Intention to Increase Physical Activity1. J Appl Soc Psychol. 2005 Sep 1;35(9):1824–48.

5. Ware J, Kosinski M, Dewey J, Gandek B. How to Score and Interpret Single-Item Health Status Measures: A Manual for Users of the SF-8 Health Survey. Boston: QualyMetric; 2008.

6. Panter J, Griffin S, Ogilvie D. Active commuting and perceptions of the route environment: a longitudinal analysis. Prev Med. 2014 Oct;67:134–40.
